# Characterization, antibacterial, and cytotoxic activities of silver nanoparticles using the whole biofilm layer as a macromolecule in biosynthesis

**DOI:** 10.1038/s41598-023-50548-9

**Published:** 2024-01-03

**Authors:** Aghapy Yermans Yakoup, Azza G. Kamel, Yasmin Elbermawy, Abdallah S. Abdelsattar, Ayman El-Shibiny

**Affiliations:** 1 Center for Microbiology and Phage Therapy, Zewail City of Science and Technology, Giza, 12578 Egypt; 2 Faculty of Environmental Agricultural Sciences, Arish University, Arish, 45511 Egypt

**Keywords:** Microbiology, Nanoscience and technology

## Abstract

Recently, multi-drug resistant (MDR) bacteria are responsible for a large number of infectious diseases that can be life-threatening. Globally, new approaches are targeted to solve this essential issue. This study aims to discover novel antibiotic alternatives by using the whole components of the biofilm layer as a macromolecule to synthesize silver nanoparticles (AgNPs) as a promising agent against MDR. In particular, the biosynthesized biofilm-AgNPs were characterized using UV-Vis spectroscopy, electron microscopes, Energy Dispersive X-ray (EDX), zeta sizer and potential while their effect on bacterial strains and normal cell lines was identified. Accordingly, biofilm-AgNPs have a lavender-colored solution, spherical shape, with a size range of 20–60 nm. Notably, they have inhibitory effects when used on various bacterial strains with concentrations ranging between 12.5 and 25 µg/mL. In addition, they have an effective synergistic effect when combined with phage ZCSE9 to inhibit and kill *Salmonella enterica* with a concentration of 3.1 µg/mL. In conclusion, this work presents a novel biosynthesis preparation of AgNPs using biofilm for antibacterial purposes to reduce the possible toxicity by reducing the MICs using phage ZCSE9.

## Introduction

In 1928, the discovery of penicillin showed its capability to prevent the growth of *Staphylococcus* bacteria^1^, and it showed a distinct effect as an antibacterial agent on other Gram-positive bacteria^2^. Henceforth, using penicillin and its derivatives could limit the number of infections to be the golden era for reducing microbial infections^3^. A resistance had risen from either mutation, misusing antibiotics, or hosting foreign DNA from the environment^4–6^. Subsequently, various bacterial species acquired resistance to the most common antibiotic and turned them into superbugs^7^. Currently, MDR bacterial infections are difficult to be cured and might be life-threatening^8^. Therefore, further research is needed to find alternatives to limit MDR infections, such as using enzymes, immunostimulants, NPs, and organic acids^9–11^. As a result, scientists are struggling to formulate new antibiotics produced from natural products, synthetic products, or a combination of both of them^12–15^. Despite using phages and probiotics as novel approaches, which are alternatives to antibiotics, they might have undesirable results, such as recurrence and resistance^16,17^.

NPs have distinctive physical and chemical properties because of their high area-to-volume ratio and small size surfaces^18,19^. Accordingly, these properties enabled them to be beneficial in numerous applications in biotechnology, including environmental remediation, medical imaging, and drug delivery^20,21^. Practically, AgNPs are widely used and studied, despite their toxicity, due to their potent antibacterial^22^, antimicrobial^23^, and antifungal properties^24^^,^^25^. Furthermore, investigations on green synthesis have emerged as a new approach in nanoscience with significant implications in the biopharmaceutical and food industries^26,27^. One of the green synthesis techniques involves the implementation of microorganisms, such as fungi, algae, and bacteria, to reduce metal ions into NPs^28–31^. These techniques have several advantages, including using natural reducing agents and the potential for synthesizing NPs with unique properties that are difficult to achieve using traditional chemical methods^32^.

One of the highly effective alternative approaches is using phage to treat MDR bacterial infections^33–35^. Phages are viruses that target only a specific bacterial species in the environment and cause bacterial cell lysis^36^. Hence, they gained an interest in various medical applications due to their high specificity without affecting normal microbiota^37–39^. For instance, topical skin infections of *Cutibacterium acnes*^40^ and acute respiratory infections^41^ are treated using phages. Despite its high specificity, bacteria can resist it after a specific duration^42,43^. Furthermore, phage resistance results from some mutations in the bacterial genetic material^44,45^, which leads to the development of new virulence factors^46,47^. Correspondingly, the resistance increases while increasing the dosage of phage in the treatment period^48,49^. Some studies found that non-immunocompromised patients produce antibodies after the first month of treatment that reduce the effectiveness of the phage therapy^50,51^. Therefore, there is an urge to find a new approach to limit the development of such resistance.

Biofilms are complex bacterial communities ensconced within an extracellular matrix to protect them against host immune responses and environmental stressors^52^. Unexpectedly, biofilm layer synthesis is cost-effective, readily available, the synthesis process is eco-friendly, and does not use toxic chemicals, which makes them more advantageous over traditional chemical methods^53^. Biomolecules of biofilm of *Pseudomonas aeruginosa *(*P. aeruginosa*)*,* such as proteins, lipids, and enzymes, are often utilized as reducing agents, independently, in the biosynthesis process of various NPs^54,55^. On the contrary, biofilm has been used in various beneficial manners for humanity, for instance, in breaking down toxic oils present in seas and oceans^56^, protecting plants’ roots from harmful bacteria^57^, and producing energy in bio-electrochemical systems while treating the used water^58,59^. Consequently, the unique combination and distinct properties of the ingredients present in the biofilm of *Pseudomonas aeruginosa *(*P. aeruginosa*) may offer benefits and advantages over the previously used biomolecules.

In this study, the biofilm layer of *P. aeruginosa* is used in the biosynthesis process of AgNPs. The outcomes of this study provide promising findings, suggesting that the ingredients present in the biofilm can effectively synthesize NPs in an eco-friendly and sustainable manner. Furthermore, the study findings may inspire further research to determine the potential of the biofilm of *P. aeruginosa* and its constituent molecules for the biosynthesis of NPs.

## Material and methods

### Materials

The materials used in this work are Tryptic Soybean Broth (TSB; MERCK, Germany), Tryptic Soy Agar (TSA; MERCK, Germany), silver nitrate (AgNO_3_; Techno Pharmachem, India) 3-(4,5-dimethylthiazol-2-yl)-2,5-diphenyltetrazolium bromide (MTT; Sigma Aldrich, US) , Dulbecco's Modified Eagle Medium (DMEM; Serana, Europe), Human Skin Fibroblast cell line (HSF), Human breast cancer cell line (MCF-7), Human liver cancer cell line (HepG2), Fetal Bovine Serum (FBS; Serana, Europe) and acidic isopropanol (Thermo fisher, US). In addition, Specific bacterial strains were used in this study to illustrate the effect of the biofilm-AgNPs. *Salmonella enterica* subsp. *enterica* serotype Typhi (*S. enterica*) NCTC 160 was grown at Xylose Lysine Deoxycholate agar (XLD; Oxoid, England), *Escherichia coli *(*E. coli*) ACTT 8739 was grown at Eosin Methylene blue (EMB; Oxoide, England), *P. aeruginosa* ACTT 27853 was grown at Cetrimide (MERCK, Germany), *Staphylococcus aureus* (*S. aureus*) ACTT 25923 was grown at Mannitol Salt Agar (MSA; Oxoide, England) and *Bacillus cereus* ACTT 14579 was grown at MYP agar (MERCK, Germany). In addition, phage ZCSE9 was used for *S. enterica* with an initial titer of 2 × 10^9^ PFU/mL.

### Methods

#### Preparation of biofilm-AgNPs

##### Preparation of the biofilm of* P. aeruginosa*

Briefly, the preparation of the biofilm of *P. aeruginosa* was executed as previously described^60^ with minor modifications. *P. aeruginosa* OL375153 was incubated in a 50 mL TSB flask at 37°C for 48 h. Then, after removing the planktonic cells, the biofilm layer was washed thrice with sterile distilled water to obtain a ring-shaped biofilm layer. The obtained biofilm was subsequently utilized for synthesizing AgNPs.

##### Biosynthesis of biofilm-AgNPs

**Figure 1:**
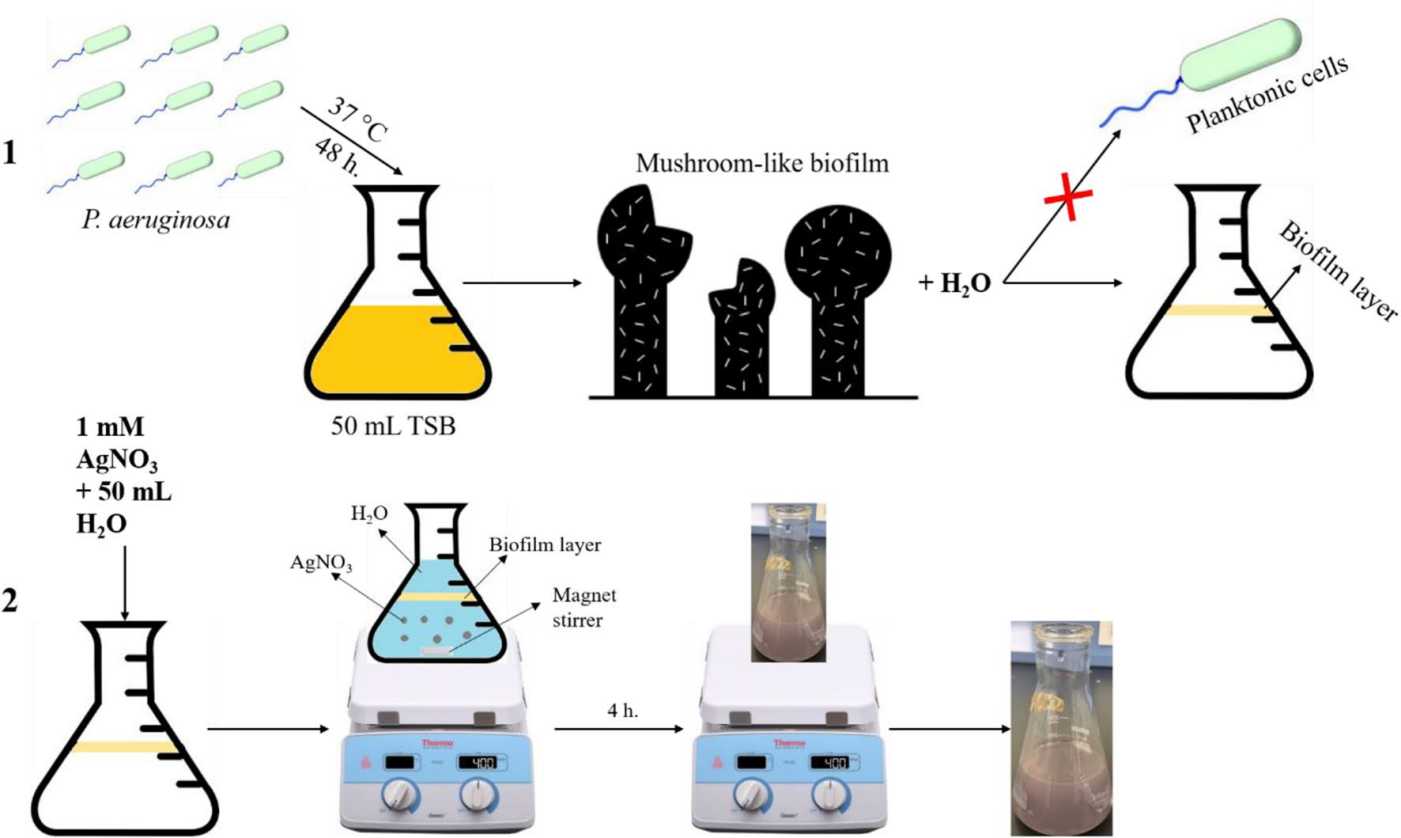
Illustrates the preparation steps (1) the preparation of biofilm layers for (2) the biosynthesis of biofilm-AgNPs.

As for the AgNPs biosynthesis process, the biofilm layer of *P. aeruginosa* was used following the previously described procedures^61^ with minor modifications. Concisely, 50 mL of 1 mM AgNO_3_ was dissolved in sterilized deionized water and provided to the biofilm. After that, the solution was stirred continuously for approximately 4 h at room temperature. Finally, the solution was prepared for further characterization, as shown in Fig. 1.

#### Biofilm-AgNPs characterization

Various tests were used to identify the characteristics of the biosynthesized biofilm-AgNPs. Scanning Electron Microscope (SEM) figure was obtained as described before^62^ by using JEOL JSM-5300, SEM (Tokyo, Japan) instrument to identify the morphology. On the other hand, Transmission Electron Microscope (TEM) was used according to the previously described protocol^63^ by applying a single drop of the sample onto carbon copper grids in a JEOL 1230, 1230 JEOL (Tokyo, Japan) to determine the size and morphology. Moreover, EDX was used to detect the percentage of the elements in the sample while relying on JEOL JSM-5300 (Tokyo, Japan) at 20 kV for a 10 mm working distance^64^. The Zeta sizer and potential of the sample were measured using rapid, non-invasive dynamin light scattering (DSL) in (Zetasizer Nano ZS (Malvern, UK)) according to^65^ with several dilutions to identify their size. The UV–Vis spectrophotometer (Jenway 7200, Staffordshire, UK) was utilized with a wavelength range of 340–800 nm^66^. The sample was not sonicated and left to dry, then take the measurement. Distilled water was utilized as a blank. Moreover, the functional groups in the biofilm-AgNPs and biofilm layers were evaluated by Fourier Transform Infrared Spectroscopy (FTIR) analysis using the Agilent system Cary 360 FTIR model, which ranges from 4000 to 400 cm^−1^.

#### Turbidity assay

A turbidity assay was conducted to identify the potential impact of the biosynthesized biofilm-AgNPs on *P. aeruginosa* described before^67^ with minor modifications. Specifically, 100 μL of biofilm-AgNPs were incubated with TSB for one night at 37 °C to evaluate the viability of *P. aeruginosa* in the presence of the biosynthesized NPs. The results were analyzed to determine whether the synthesized biofilm-AgNPs have any antibacterial properties against *P. aeruginosa*.

#### Antimicrobial analyses

##### Disc and well diffusion assays

As for testing the antibacterial effect of the biosynthesized biofilm-AgNPs, disc and well diffusion tests were performed according to Iqbal et al. and Perveen et al. with minor modifications^68,69^. Initially, each bacterium was swabbed on TSA plate using a sterilized cotton swab. Then, each plate contained two sterilized filter-paper discs with a diameter of 6 mm and two induced wells with a diameter of 6 mm. After that, one disc was loaded with 10 µL, and one well was loaded with 20 µL of the 200 µg/mL of biofilm-AgNPs while the rest were loaded with double distilled water as a negative control. Moreover, the same volumes of 1 mM AgNO_3_ serve as positive controls. Finally, the prepared plates were dried and incubated overnight at 37 °C. The antimicrobial activity of biofilm-AgNPs was evaluated by measuring the inhibition zone around the discs or the wells as an indication of killing the bacteria.

##### MIC and MBC

Furthermore, antibacterial activity was determined using the microdilution method to undergo a minimum inhibitory concentration (MIC) test according to bakht Dalir et al.^70^ with some modifications. MIC was applied in a sterilized 96-well flat-bottom plate where the biofilm-AgNPs were diluted 1:1 v/v with the bacteria to start with 200 μg/mL of biofilm-AgNPs as a final concentration. Firstly, each bacterium was cultured at 37 °C in TSB to be used with initial titer as stated: *S. enterica* was 2.3 × 10^6^ CFU/mL, *E. coli* was 1.8 × 10^6^ CFU/mL, *P. aeruginosa* was 1.9 × 10^6^ CFU/mL, *S. aureus* was 1.3 × 10^7^ CFU/mL, and *Bacillus cereus* was 4 × 10^5^ CFU/mL. Various controls were identified either by adding only bacteria or sterilized TSB. Secondly, the 96-well plate was incubated overnight at 37 °C. Then, the minimum bactericidal concentration (MBC) test is managed according to Loo et al.^71^ with modifications. First, the MBC test was applied by determining the clear or semi-clear wells in the previously prepared 96-well plate, and then 10 µL were taken from each of them to be cultivated on a TSA plate incubated at 37 °C overnight.

##### Time-Kill assay

In addition, the time-killing curve assay was measured regarding the methodology according to Abdelsattar et al.^72^ with modifications. The assay was performed by inoculating the bacteria in a 96-well plate with initial titer as stated: *S. enterica* was 2.3 × 10^6^ CFU/mL, *E. coli* was 1.8 × 10^6^ CFU/mL, *P. aeruginosa* was 1.9 × 10^6^ CFU/mL, *S. aureus* was 1.3 × 10^7^ CFU/mL, and *Bacillus cereus* was 4 × 10^5^ CFU/mL. After that, biofilm-AgNPs were added with various concentrations of 100, 50, 25, 12.5, 6.3, 3.1 and 1.6 µg/mL. Then, their growth rate was identified at 37 °C through reading OD_600_ by a microplate reader (FLUOstar Omega, BMG LABTECH, Ortenberg, Germany). The readings and the extracted data were collected every 30 min throughout 11 h using the MARS Data Analysis Software package (version 3.42). The heatmap was created using Microsoft Excel Spreadsheet.

##### MTT assay

Finally, the MTT assay was applied on a 96-well plate with the bacteria incubated with biofilm-AgNPs overnight at 37 °C according to Makky et al.^73^ with modifications. First, each well was provided with 10 µL of MTT solution (5mg/mL) then the plate was incubated while shaking for 50 min at 37 °C. Second, 100 µL of acidic isopropanol 1.5% (v/v) was mixed into each well in the plate then it was incubated again while shaking for 50 min at 37 °C. Finally, the optical density of the bacteria was measured at OD_570_ by FLUOstar Omega Microplate Reader.

#### Cell membrane integrity

Cell membrane integrity test was applied to *S. enterica* in combination with ZCSE9 according to Townsend et al.^74^ with minor modifications. *S. enterica* was cultured in TSB at 37 °C for 10 min with ZCSE9 and biofilm-AgNPs with a concentration of 12.5 µg/mL. After that, 2.5% uranyl acetate stain was used to stain the whole sample, and then it was put on a carbon-coated Cu-grid until it dried. Finally, the sample was examined using TEM (1230 JEOL, Tokyo, Japan) at different magnification powers.

#### Cytotoxicity of biofilm-AgNPs

Cell viability for the HSF cell lines was identified by MTT assay as described previously according to Yan et al.^75^ with numerous modifications. First, HSF cells (passage 8) were seeded in sterilized 96 well plates with a density seeding of (8000 cells/well) in a complete DMEM medium with 10% FBS and 100 mg/mL of streptomycin for 24 h at 37 °C at a 5% CO_2_ incubator. After that, the serial concentrations of AgNPs were added, from the range 0.1 μg/mL to 16 μg/mL, negative control without treatment, and incubated for 24 h. Then, all the wells were provided with 100μL of (MTT reagent+DMEM medium), and cells were incubated for four hours at 37°C. Finally, the medium was discarded, and 100μL of DMSO was added and measured at OD_570_.

#### Analytical statistics

The data obtained from cytotoxicity was the means of two biological replications of three technical replications, and the standard deviation (SD) was determined as the error bar. Additionally, all microscopic, Zeta and EDX graphs were obtained three times at least with different fields to ensure the presented data is the average. Moreover, GraphPad Prism v5 software was used to produce the graphs and conduct all statistical analyses.

## Results

### Biofilm-AgNPs characterization

#### Color change, UV-Vis spectrum and FTIR of biofilm-AgNPs

**Figure 2:**
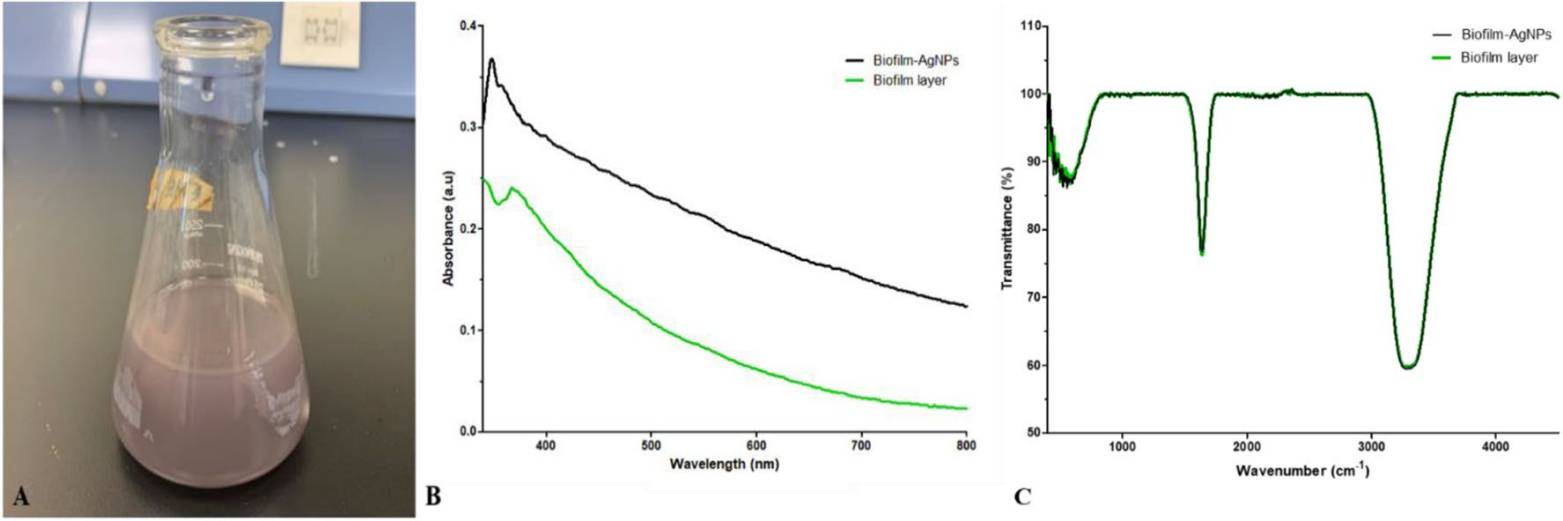
Shows (**A**) the final color of biosynthesized biofilm-AgNPs and (**B**) the UV-Vis visible spectra of biofilm-AgNPs and biofilm layers, (**C**) the FTIR for biofilm-AgNPs and biofilm layers.

The observable change of color of the prepared solution from a cloudy silver to a lavender was observed after four hours without heat, as shown in Fig. 2A. The alteration in color indicates the successfulness of reducing Ag^+^ to Ag^0^ in the AgNO_3_ solution and the success in the biofilm-AgNPs formation. Using the UV-Vis spectrophotometer indicates the possibility of the synthesized biofilm-AgNPs in the solution. The UV-Vis spectrum of the solution reveals a single peak of a maximum absorption rate at 364 nm, as shown in Fig. 2B. The FTIR was also conducted, and the results showed the same peaks for both the biofilm layer and biofilm-AgNPs. The resulting peaks at 1637.7 (for the biofilm layer) and 1637.51 (for biofilm-AgNPs) indicated the presence of the double bonds of C=O amides. However, the peaks at 3287.67 and 3283.81 indicated the presence of H–O bond in alcohols and phenolic compounds for both the biofilm layer and biofilm-AgNPs, respectively^76,77^.

#### TEM and SEM

**Figure 3:**
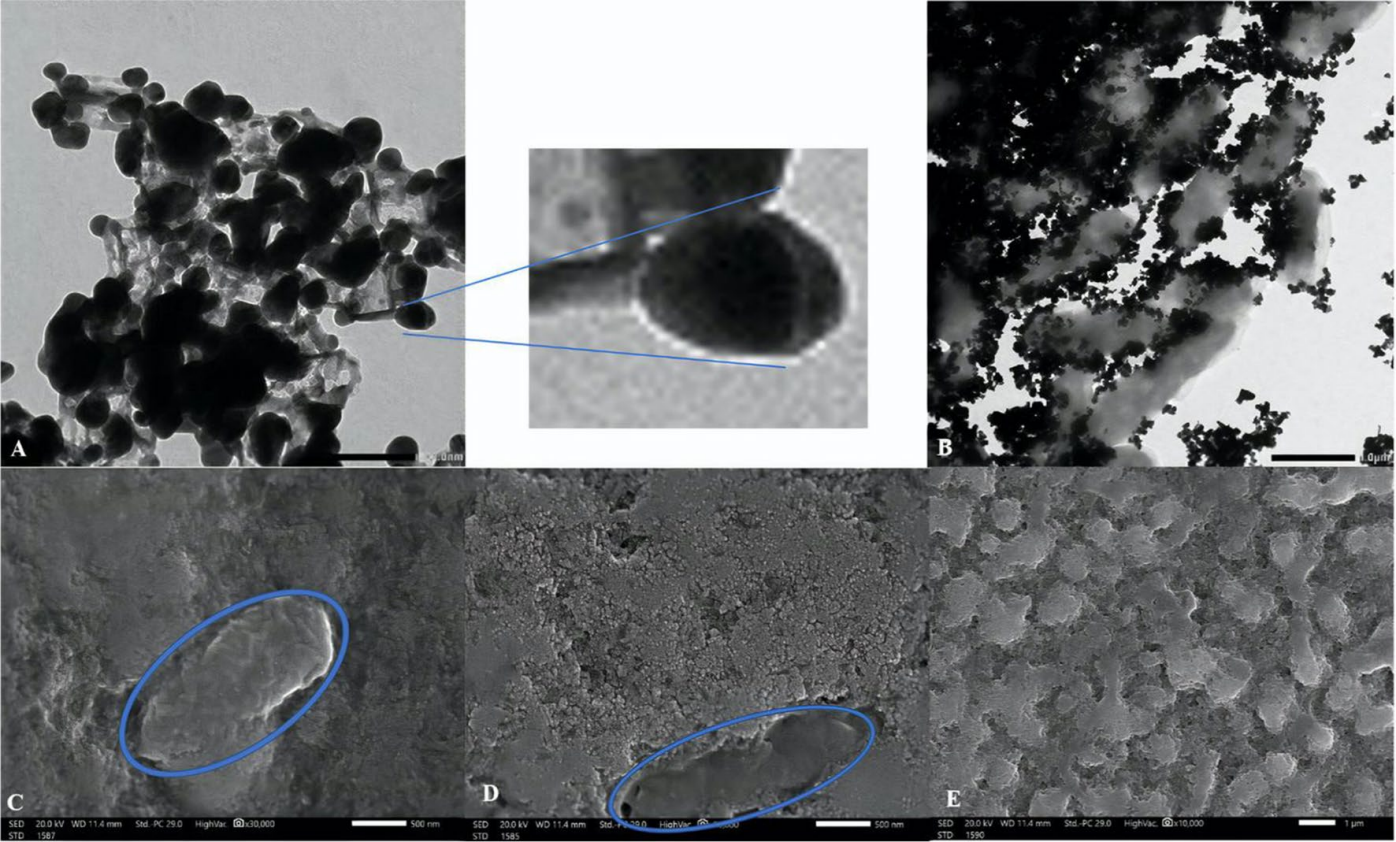
Illustrates TEM images of spherical AgNPs attached to ghost *P. aeruginosa,* where (**A**) shows the spherical structure of the formed NPs with a scale bar of 100 nm, and (**B**) shows its combination with the bacterial ghost with a scale bar of 1 µm. Moreover, SEM images of biofilm-AgNPs after their biosynthesis in combination with the bacterial ghosts in blue circles where (**C**), (**D**) with a scale bar of 500 nm, and (**E**) with a scale bar of 1 µm.

TEM and SEM results are presented for the biosynthesized biofilm-AgNPs as shown in Fig. 3. TEM images reveal the formation of spherical AgNPs with a size range from 20 to 60 nm in length, as shown in Fig. 3A. In addition, they showed that biofilm-AgNPs are surrounded with the bacterial ghost in the biofilm as shown in Fig. 3B. SEM images indicate that biofilm-AgNPs are formed in the solution with the spherical shape around the bacterial ghost cells with damaged cells wall as shown in Fig. 3C and D. Moreover, these damages in the ghost bacterial cells and rupture are shown obviously in Fig. 3E where there is a disruption in the biofilm environment. Potentially, these results highlight their ability to penetrate the biofilm layer and their interaction with its components.

#### EDX and zeta sizer and zeta potential

**Figure 4:**
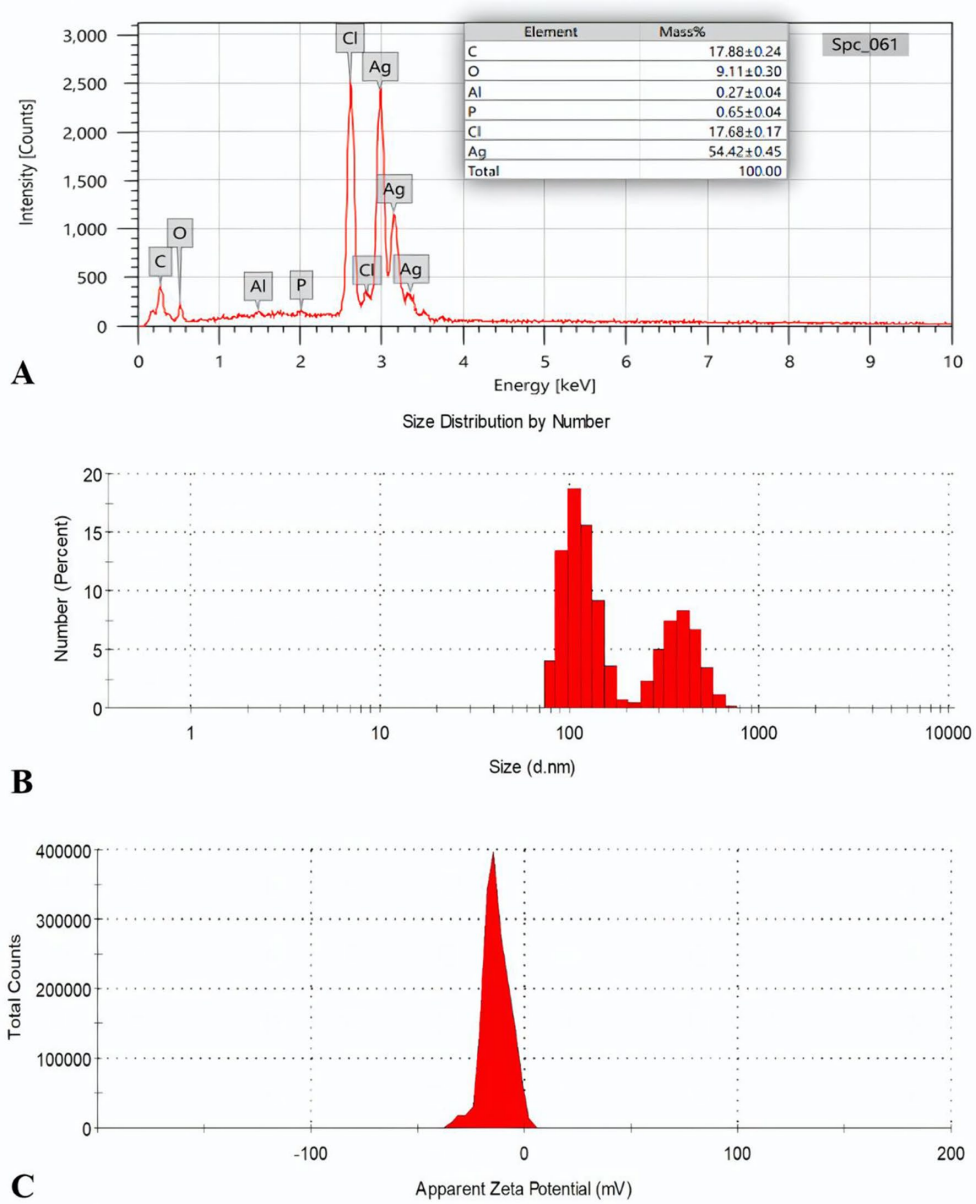
Shows (**A**) EDX spectrum analysis of biofilm-AgNPs, (**B**) Zeta sizer of AgNPs with two peaks, and (**C**) Zeta potential equal to − 19.1 mV.

The utilization of EDX enables many investigations into the metallic nature of the biosynthesized biofilm-AgNPs. The resulting EDX spectrum showcases a strong signal at 3 keV, thereby indicating the successful synthesis of AgNPs, as shown in Fig. 4A. Additionally, the EDX spectrum indicates the presence of Ag with percentage of 54.42%, carbon (C) with 17.88%, chlorine (Cl) with 17.68%, oxygen (O) with 9.11%, phosphorus (P) with 0.65% and aluminum (Al) with 0.27%. The analysis of surface charge exhibited that biofilm-AgNPs have a net negative charge. Moreover, the investigation of the biofilm-AgNPs on Zeta Potential shows a sharp peak at -19.1 mV, as shown in Fig. 4C. As the high negative or positive charge refers to the repulsion between the particles and the low aggregation level, it was suggested that the biofilm-AgNPs had a relatively low aggregation than other AgNPs preparations ^78^. In addition, the Zeta Sizer analysis of the biofilm-AgNPs indicates their uniformity of sizes within the range of 100 to 1000 nm, as shown in Fig. 4B. The size range shows two different peaks with two different ranges. The first peak is from 70 to 200 nm, and the most dominant size for this peak was 100 nm. Moreover, the second peak ranged from 200 to 700 nm, and the most dominant size in this peak was 400 nm for the prepared NPs.

### Antimicrobial tests

#### Bacterial results

##### Disc and well diffusion tests

**Figure 5:**
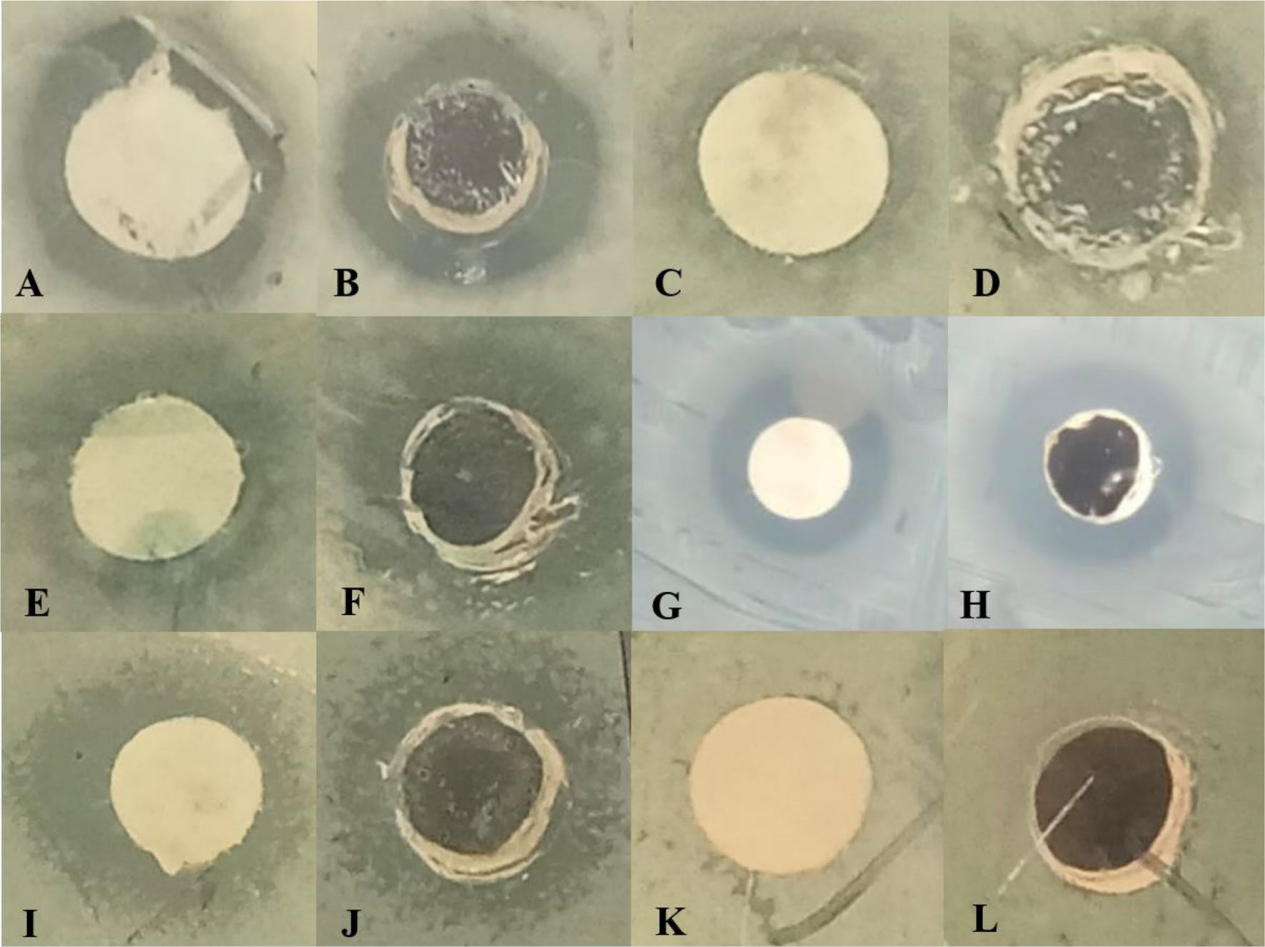
Shows the effect of biofilm-AgNPs when tested on several bacterial strains where (**A** and **B**) for *Bacillus cereus*, (**C** and **D**) for *E. coli*, (**E** and **F**) for *P. aeruginosa*, (**G** and** H**) for *S. aureus*, (**I** and **J**) for *S. enterica* and (**K** and **L**) for negative controls.

**Figure 6:**
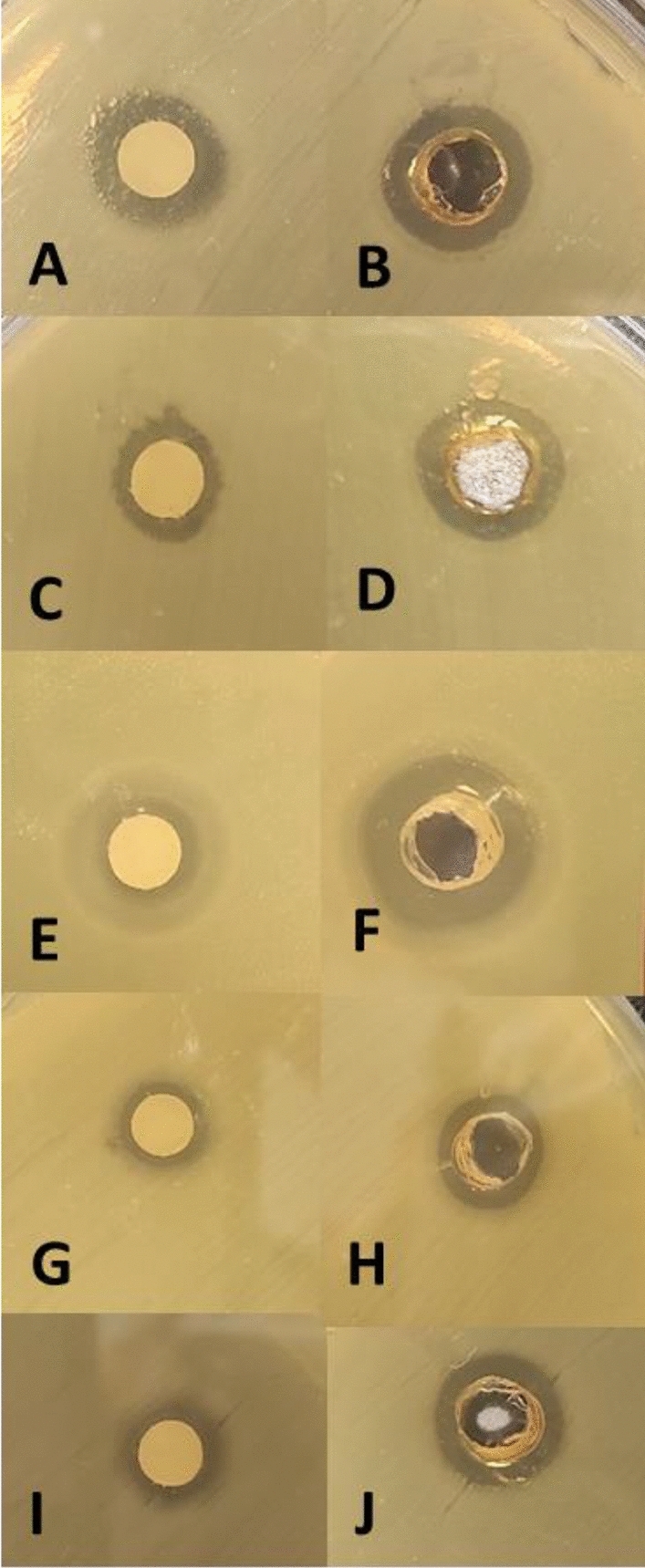
Shows the effect of positive control (silver nitrate) when tested on the same bacterial strains where (**A** and **B**) for *Bacillus cereus*, (**C** and **D**) for *E. coli*, (**E** and **F**) for P. aeruginosa, (**G** and **H**) for S. aureus, (**I** and **J**) for S. enterica.

Disc and well diffusion tests were performed to detect the primary antibacterial activity of biosynthesized biofilm-AgNPs and Silver nitrate (positive control) on various bacterial stains, as shown in Figs. 5 and 6. The results represented in Table 1 illustrate the presence of an obvious antimicrobial activity for the biofilm-AgNPs with a concentration of 200 µg/mL. The highest effect of disc diffusion tests based on inhibition zones was 12.2 mm for *S. enterica* 10.7 mm for *S. aureus*, 10.4 mm for *Bacillus cereus,* 9.7 mm for *P. aeruginosa* and 8.9 mm for *E. coli*. The highest effect of well diffusion tests based on inhibition zones was 13.3 mm for *S. aureus*, 12.2 mm for *Bacillus cereus,* 11.8 mm for *P. aeruginosa*, 11.8 mm for *S. enterica* and 8.9 mm for *E. coli*. The inhibition zones were compared to the positive and negative controls.

**Table 1:** Represents the results of the disc and well diffusion tests for the biofilm-AgNPs compared to the positive control, in addition to those of MIC and MBC when biofilm-AgNPs were subjected to the used bacterial strains.

	Disc and well diffusion results biofilm-AgNPs		Disc and well diffusion results for positive control		MIC and MBC results	
	Disc (mm)	Well (mm)	Disc (mm)	Well (mm)	MIC (µg/mL)	MBC (µg/mL)
*B. cereus*	10.4	12.2	10	11	25	25
*E. coli*	8.9	8.9	9.5	12	25	100
*P. aeruginosa*	9.7	11.8	14	15	100	200
*S. aureus*	10.7	13.3	11	10.9	12.5	100
*S. enterica*	12.2	11.8	85	9.8	25	100
*S. enterica*+ZCSE9	No results				3.1	3.1

##### MIC and MBC

Accordingly, MIC and MBC tests were applied to detect the effect of biofilm-AgNPs on these previously used bacterial strains. As shown in Table 1, the prepared biofilm-AgNPs have a clearance effect on the used bacteria with various concentrations. The lowest effect on MIC tests was 12.5 µg/mL for *S. aureus*, 25 µg/mL for *Bacillus cereus* and *E. coli,* and 100 µg/mL for *P. aeruginosa*. The lowest effect on MBC tests was 25 µg/mL for *Bacillus cereus*, 100 µg/mL for *S. aureus* and *E. coli,* in addition to 200 µg/mL for *P. aeruginosa*. The inhibitory and bactericidal effects were compared to several controls mentioned before.

##### Time-killing curve

**Figure 7:**
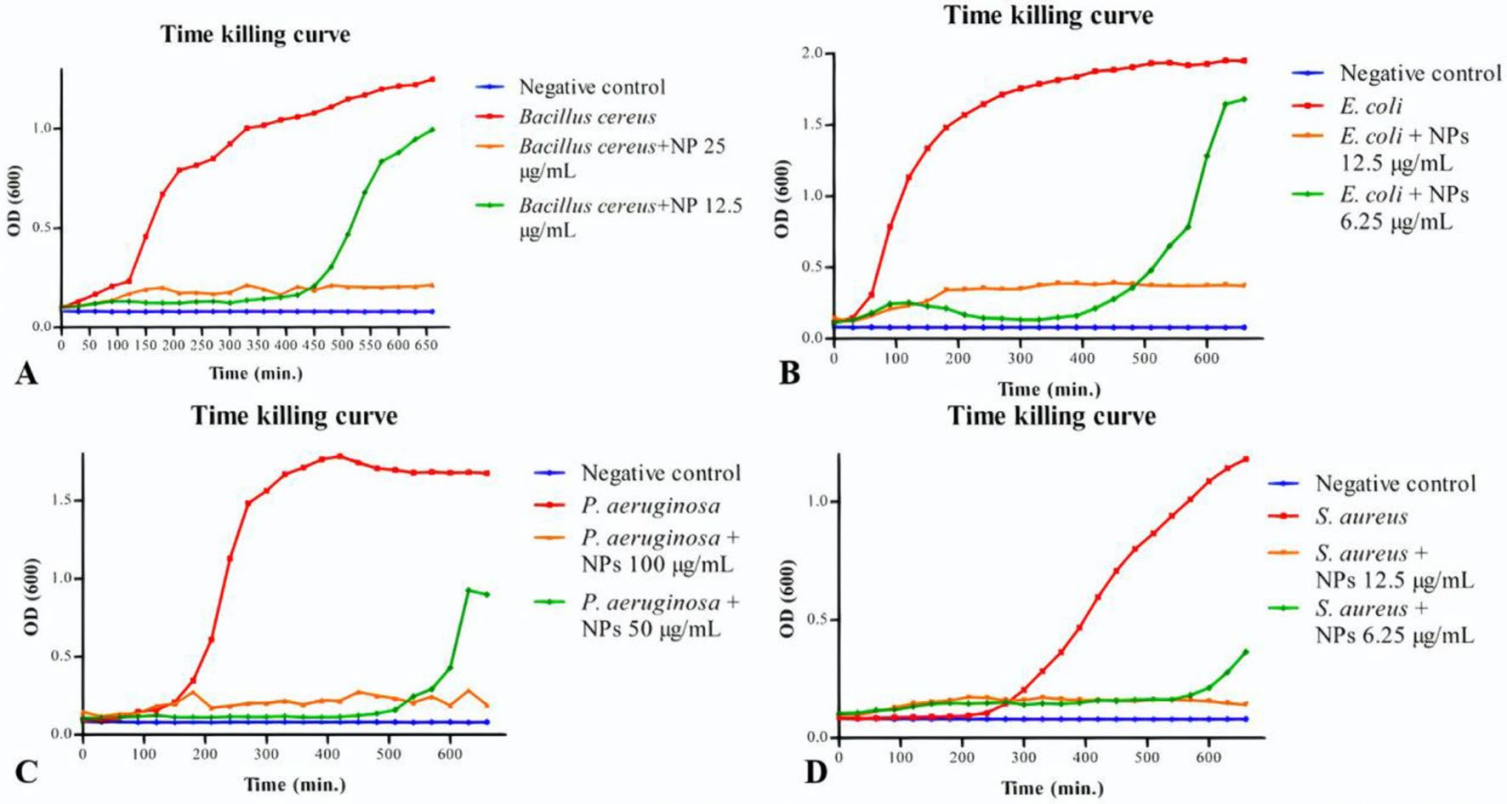
Illustrates time-killing curves of the used bacterial strains to detect their growth rate under the effect of biofilm-AgNPs where (**A**) is *Bacillus cereus* graph, (**B**) is *E. coli* graph, (**C**) is *P. aeruginosa* graph, and (**D**) is *S. aureus* graph.

**Figure 8:**
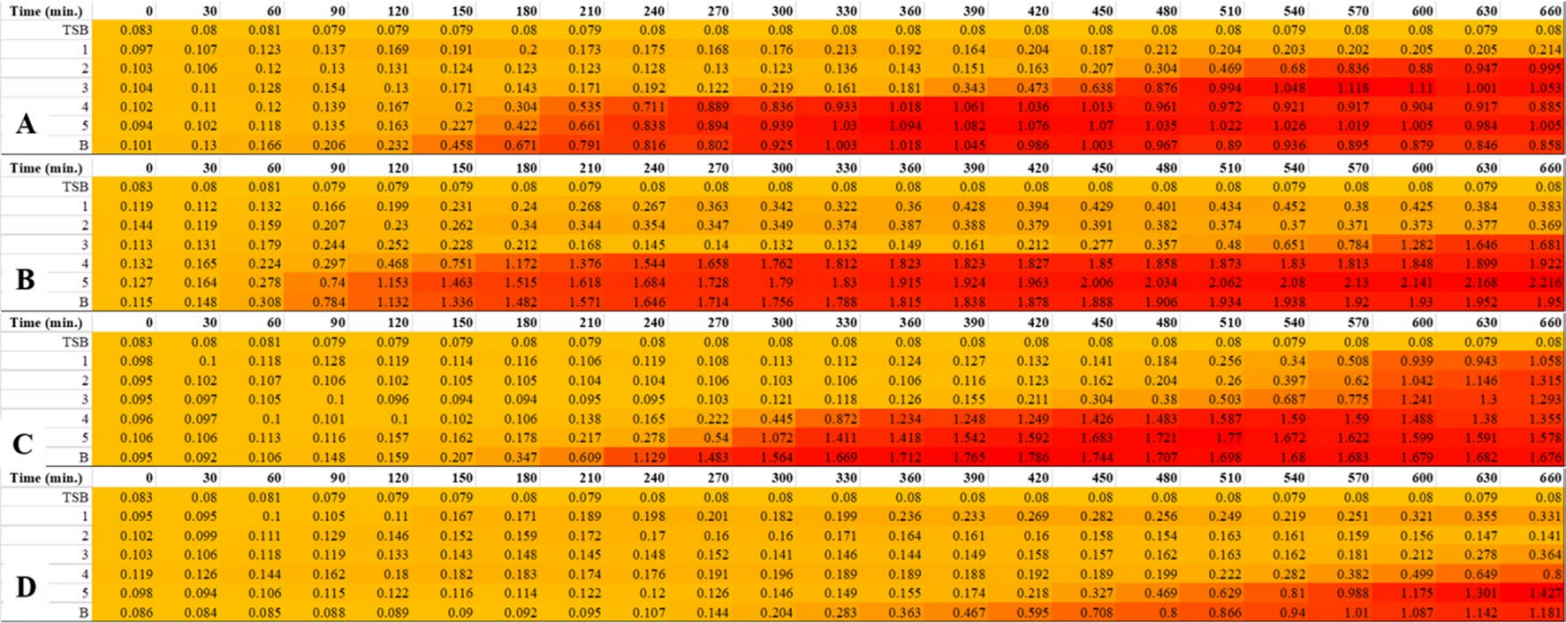
Illustrates a heat map for the selected bacterial strains that indicates their growth under the effect of biofilm-AgNPs where the golden yellow color indicates low concentration while the red color indicates high concentration. (**A**) is *Bacillus cereus*, (**B**) is *E. coli*, (**C**) is *P. aeruginosa*, and (**D**) is *S. aureus*. The numbers 1, 2, 3, 4 & 5 represents the concentrations of biofilm-AgNPs 25, 12.5, 6.3, 3.1 and 1.6 µg/mL, respectively, and (**B**) is the bacteria without any treatment. The heatmap was created using Microsoft Excel Spreadsheet.

Time-killing curve was conducted to detect a spectrum for bacterial growth under the effect of biofilm-AgNPs, and the variation in bacterial optical density (OD_600_), as shown in Fig. 7. These results were confirmed by the heat maps represented in Fig. 8, which shows the turbidity of the bacteria along 660 min continuously with golden yellow color for the less turbid wells and red color for the highly turbid wells. Several controls were applied, which are empty TSB wells shown as the blue line and bacterium without any treatment shown as the red line. At the concentration of 6.25 µg/mL, *E. coli* was inhibited for 420 min., and *S. aureus* was inhibited for 570 min. At the concentration of 12.5 µg/mL, *Bacillus cereus* was inhibited for 420 min., and E*. coli* and *S. aureus* were inhibited for 660 min. At the concentration of 25 µg/mL, *Bacillus cereus* was inhibited for 660 min. At the concentration of 50 µg/mL, *P. aeruginosa* was inhibited for 510 min. At the concentration of 100 µg/mL, *P. aeruginosa* was inhibited for 660 min*.*

#### *S. enterica* with ZCSE9 results

##### MIC and MBC

MIC and MBC tests were applied to detect the effect of biofilm-AgNPs on *S. enterica* when it is combined with ZCSE9. As shown in Table 1, the prepared biofilm-AgNPs have a tangible effect on *S. enterica.* The lowest MIC effect was 3.1 µg/mL for *S. enterica* combined with ZCSE9, while it was 25 µg/mL for *S. enterica* alone. The lowest MBC effect was 3.1 µg/mL for *S. enterica* combined with ZCSE9, while it was 100 µg/mL for *S. enterica* alone.

##### Time-killing curve

**Figure 9:**
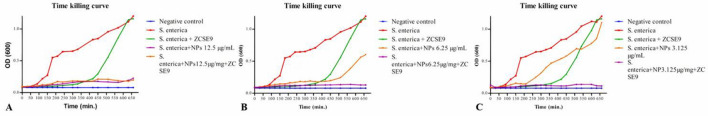
Illustrates time killing curves *S. enterica* to detect its growth rate under the effect of biofilm-AgNPs with and without the presence of ZCSE9 where A is the NP with concentration 3.1 µg/mL, B is the NP with concentration 6.3 µg/mL, and C is the NP with concentration 12.5 µg/mL.

**Figure 10:**
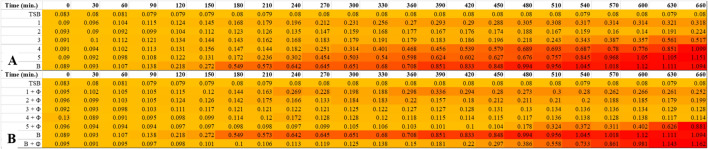
Illustrates heat maps for the selected bacterial strains that indicate their growth under the effect of biofilm-AgNPs, where the golden yellow color indicates low concentration, and the red color indicates high concentration. (**A**) is *S. enterica* with the NPs alone, while (**B**) is *S. enterica* with the NPs in combination with ZCSE9. The numbers 1, 2, 3, 4 and 5 represent the concentrations of biofilm-AgNPs 25, 12.5, 6.3, 3.1 and 1.6 µg/mL, respectively. (**B**) is the bacteria without any treatment, and (Φ) is ZCSE9. The heatmap was created using Microsoft Excel Spreadsheet.

A time-kill curve was conducted to detect the spectrum for *S. enterica* growth under the influence of biofilm-AgNPs alone and when combined with ZCSE9, the variation in bacterial optical density (OD_600_) as shown in Fig. 9. Several controls were applied which are empty TSB wells shown as the blue line and bacterium without any treatment shown as the red line. These results were confirmed by the heat maps represented in Fig. 10, which shows the turbidity of the bacteria for 11 h continuously, with a golden yellow color for the less turbid wells and a red color for the highly turbid wells. At the concentration of 12.5 µg/mL, *S. enterica* alone and in combination with ZCSE9 were inhibited for 660 min. At the concentration of 6.3 µg/mL, 11 h is needed to inhibit *S. enterica* in combination with ZCSE9 was inhibited for 660 min. and *S. enterica* only was inhibited for 480 min. At the concentration of 3.1 µg/mL, *S. enterica,* in combination with ZCSE9, was inhibited for 660 min, and *S. enterica* was inhibited for 240 min.

### Cell membrane integrity

**Figure 11:**
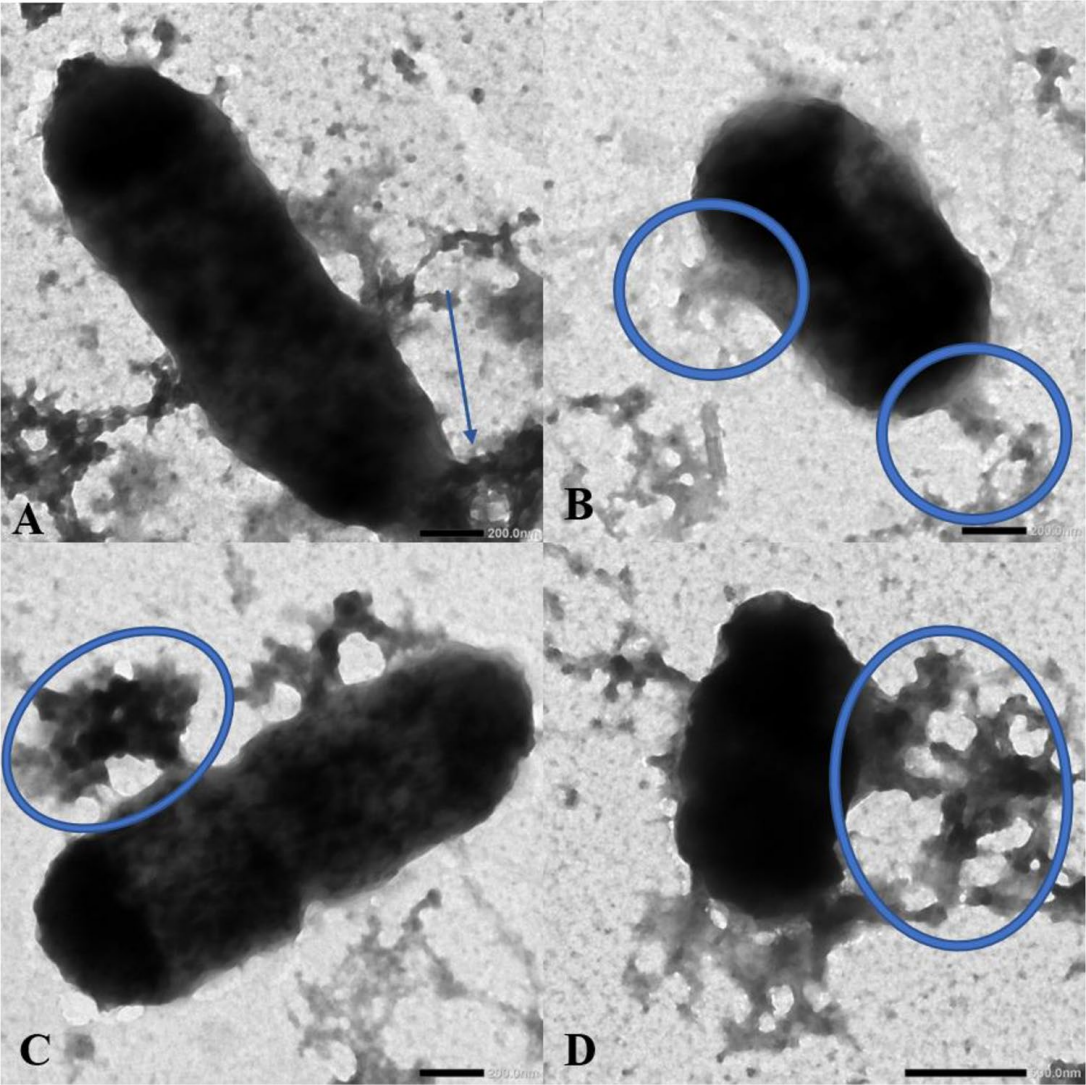
Illustrates that using biofilm-AgNPs with 0.5 × MIC concentration with ZCSE9 towards *S. enterica* leads to disruption in the cell membrane, and the intracellular components come out of the cell as shown in the blue circles and arrow where the scale bar of (**A**), (**B**), and (**C**) was at 200 nm while (**D**) was at 500 nm.

The results of the integrity of bacterial cell membranes were illustrated using a TEM analysis device, as shown in Fig. 11. Incubating *S. enterica* with biofilm-AgNPs with a concentration of 0.5 MIC and ZCSE9 had various effects on the cell membrane integrity. Biofilm-AgNPs, in combination with ZCSE9, lead to disruption in the cell membrane, which leads to rupture in some parts, and the intracellular components come out of the cell, as shown in the blue circles and arrow in Fig. 11.

### Cytotoxicity of biofilm-AgNPs

**Figure 12:**
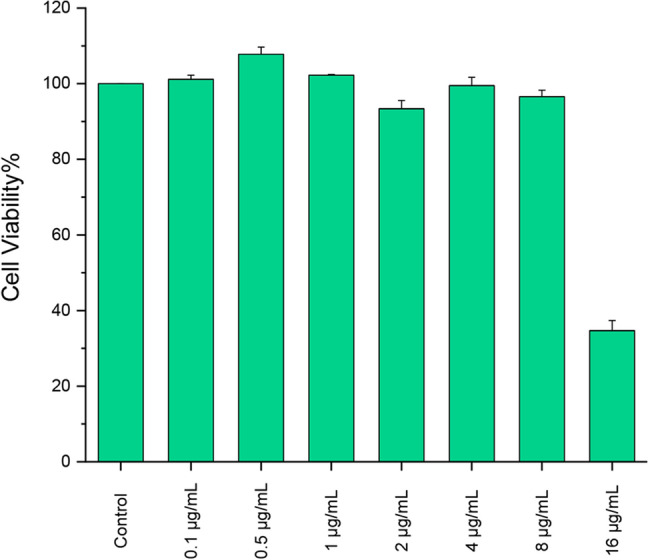
Represents the cytotoxicity of biofilm-AgNPs on the HSF cell line at various concentrations after exposure.

To assess the safety of biofilm-AgNPs, a cytotoxicity assay has been conducted on the HSF cell line with various concentrations. The results have shown in Fig. 12 that biofilm-AgNPs are not toxic and do not cause damage when exposed to the HSF cell line at concentrations 0.1 μg/mL to 8 μg/mL. When increasing the concentration to 16 μg/mL, it reduced the cell viability to 34.70%.

## Discussion

In 2022, the WHO reported that infection rates are continuously increasing. Statistically, more than 50% of bacterial infections around the world are due to MDR bacteria^79^. A long time ago, these MRD bacteria could transform, directly or indirectly, and cause a series of illnesses in humans and animals, such as *S. aureus*, *E. coli*, *S. enteric*, *Bacillus cereus*, and *P. aeruginosa*^80–83^. For instance, these reports demonstrated that more than 20% of clinically isolated *E. coli* are resistant to the first and second lines of treatments^79,84^. Therefore, tackling new approaches to treat or defeat MDRs is one of the universal goals^85^. In this study, biosynthesized NPs, biofilm-AgNPs, were prepared as a new approach to determine their antibacterial effect on the previously mentioned bacteria. Ag is the metallic ion used to synthesize the NPs due to its well-known antibacterial activity^86,87^. It is found that green synthesized and biosynthesized NPs have a higher antimicrobial effect than metallic ones^88–90^. In this study, the biofilm of *P. aeruginosa* is used in the biosynthesis process of AgNPs in a trial to determine its effect. In previous studies, some components of the biofilm layer were used as reducing agents in synthesizing NPs, such as extracellular polymer substances^91^, intracellular polymeric substances^92^ and various enzymes such as dehydrogenases^93^. Although these components were used independently, it is the first time to use all of the components altogether. It is hypothesized that using the whole biofilm layer might enhance the antimicrobial activity of the AgNPs.

The biosynthesized biofilm-AgNPs were prepared according to the steps illustrated in Fig. 1 and then characterized. There was a clear change in the color of the previously prepared solution from a cloudy silver color to a lavender color. This change occurred after four hours of continuous stirring for the solution without heat which is considered as a primary indicator for the synthesis of the NPs. According to previous studies, AgNPs can have various ranges of colors according to the natural product or substance used during its synthesis^94,95^. They can have brown, red, green or other rainbow colors^96,97^. The change in the color of the solution signifies the presence of a redox reaction where Ag^+^ is reduced to Ag^0^ to form NPs^98,99^. After the color change, UV-Vis spectroscopy analysis for the prepared solution identified a single peak of wavelength at 364 nm. Although previous studies had mentioned that the surface plasmon resonance (SPR) absorption of AgNPs ranges between 400 and 450 nm^100–102^, some studies documented that using a biological reducing agent will shift the peak to less wavelength range^103,104^. TEM and SEM results illustrated that the size of biofilm-AgNPs ranges between 20 and 60 nm with a spherical shape. These results are similar to previous studies where AgNPs have a size range of 20–90 nm with a spherical shape^105–107^. Here, it is suggested that the link between the biofilm components reduced the size of the formed NPs according to what was mentioned before^108^. As illustrated in Fig. 3, it is suggested that Ag^+^ could rupture the bacterial cells and penetrate the biofilm layer to use its components in the biosynthesis of the NPs. This might indicate its antibacterial effect, which is supported by previous studies that used bacteria in synthesizing NPs^109,110^. EDX results demonstrate the elemental analysis of the prepared sample. As presented in Fig. 4, about 54% of the sample contains Ag, while some elements represent the main components of natural products, such as carbon, with 17.88% and oxygen, with 9.11%. As detected in previous studies, these are the main components found in the biosynthesized NPs^111,112^. The results also show the presence of chlorine, which is represented as a source of contamination. The results of zeta illustrate the successful preparation and the possibility of being functional and stable. When compared to other studies, it is suggested that the more negative result might indicate more effect when applied on bacteria^113–115^. In addition, the zeta sizer illustrated two populations; it was suggested that the first population refers to the biofilm-AgNPs alone, and the second population refers to the biofilm-AgNPs bound to the *P. aeruginosa*. This finding is compatible with the results from TEM and SEM analysis (Fig. 3).

**Figure 13:**
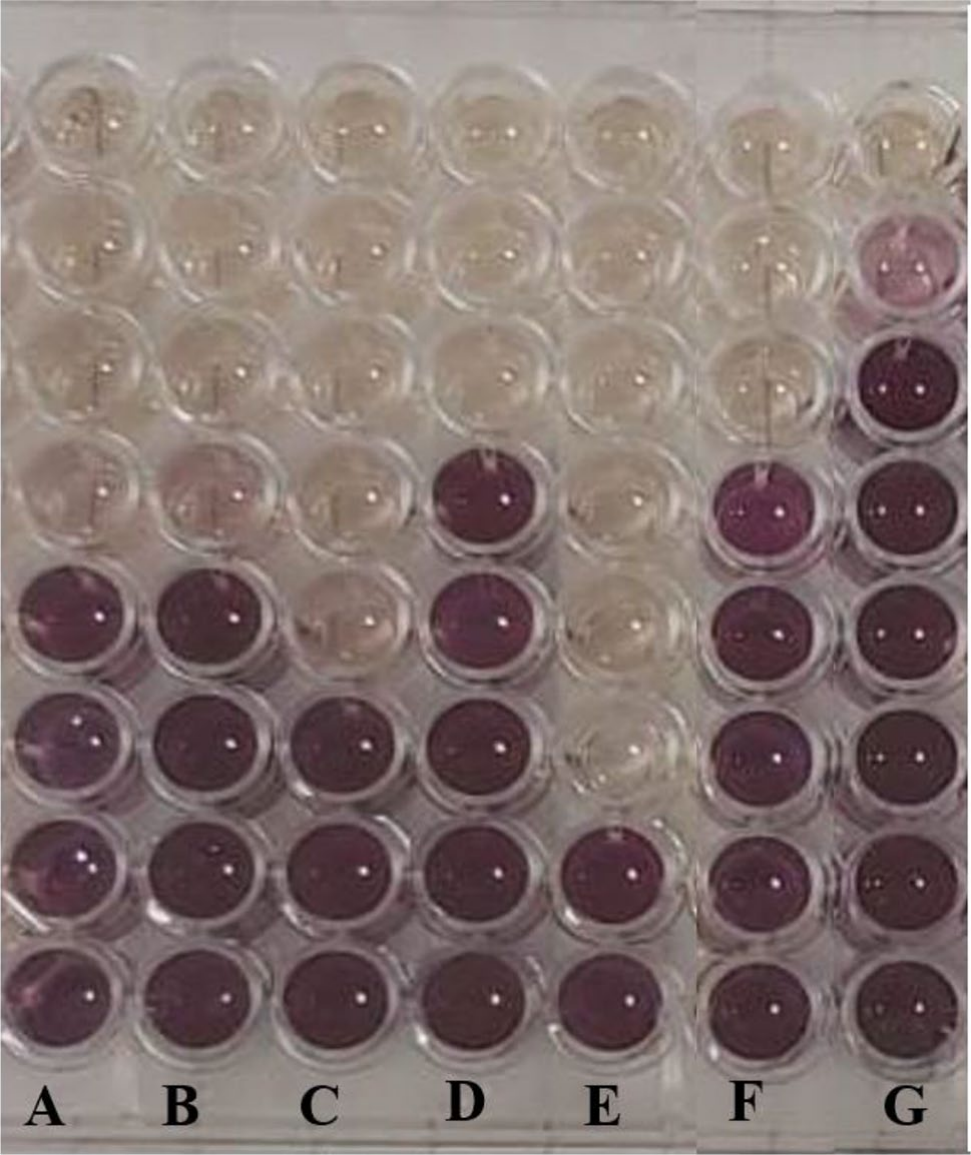
Shows the colorimetric change after using the MTT assay lanes containing (**A**) *S. aureus,* (**B**) *E. coli*, (**C**) *S.* Typhimurium, (**D**) *S. enterica*, (**E**) *S. enterica*+ZCSE9, (**F**) *Bacillus cereus*, and (**G**) *P. aeruginosa.* In addition, the rows from 1 to 8 contain biofilm-AgNPs with concentrations from (100, 50, 25, 12.5, 6.25, 3.1, 1.6 and 0.8 µg/mL), respectively.

Biofilm-AgNPs were tested on Gram-positive bacteria, such as *Bacillus cereus* and *S. aureus*, and Gram-negative bacteria, such as *E. coli, S. enterica, and P. aeruginosa.* Previous studies demonstrate that AgNPs have a strong antibacterial effect on Gram-positive and Gram-negative bacteria^116–118^. The disc and well diffusion test results showed that the inhibitory zones vary on Gram-positive and Gram-negative bacteria when biofilm-AgNPs were used with a concentration of 200 µg/mL. Afterward, MIC results highlighted that biofilm-AgNPs have the lowest inhibitory effect of 12.5 µg/mL for *S. aureus,* 25 µg/mL *E. coli*, *Bacillus cereus* and *S. enterica* and 100 µg/mL for *P. aeruginosa*. Besides, they have the lowest bactericidal effect of 25 µg/mL for *Bacillus cereus*, 100 µg/mL for *E. coli*, *S. aureus* and *S. enterica*, 200 µg/mL for *P. aeruginosa*. Yet, when biofilm-AgNPs were combined with ZCSE9, which is specific for *S. enterica*^119^, its inhibitory and bactericidal effects were enhanced to have a higher effect in lower concentration, which is 3.1 µg/mL. These results were confirmed using a killing curve, which showed that when using biofilm-AgNPs with its MIC, it would inhibit the growth of the bacteria for up to 11 h. This long period of time might indicate the long-lasting effect of the biofilm-AgNPs. This might enable them to be used as a detergent or a disinfectant for surfaces in hospitals and other places. Studies show that the effectiveness of the daily used detergents ranges from a few minutes to a few hours^120–122^. When biofilm-AgNPs are used with a concentration less than the bacterial MIC, 12.5 µg/mL for *Bacillus cereus*, *E. coli,* and *S. enterica*, 6.3 µg/mL for *S. aureus* and 50 µg/mL for *P. aeruginosa*, the inhibitory effect will last for less time but still effective at inhibiting their growth. These results were confirmed with the MTT assay, as shown in Fig. 13, in which the MTT assay was conducted to confirm the antibacterial effect of biofilm-AgNPs. The obvious effect of biofilm-AgNPs is conducted from the ability of AgNPs to penetrate the bacterial cell membrane and destroy it^123,124^. Its accessibility to the membrane indicates that the effect on Gram-negative bacteria is more than the effect on Gram-positive ones^125,126^. In this study, it is suggested that AgNPs affect various bacterial strains by producing ions that interact with cellular organelles, causing their damage^127–129^. Several studies explained the mechanism based on breaking down the cell membrane, entering the cell through porin proteins, disrupting mitochondrial function, generating Reactive Oxygen Species (ROS), breaking apart ribosomes, denaturing proteins, stopping ATP production and causing damage to DNA^14,130–133^. Here, we suggest that one of the mechanisms of biofilm-AgNPs could be a result of attaching NPs to the cell membrane and then interrupting bacterial selective permeability, causing the release of cellular contents, as in Fig. 11.

**Figure 14:**
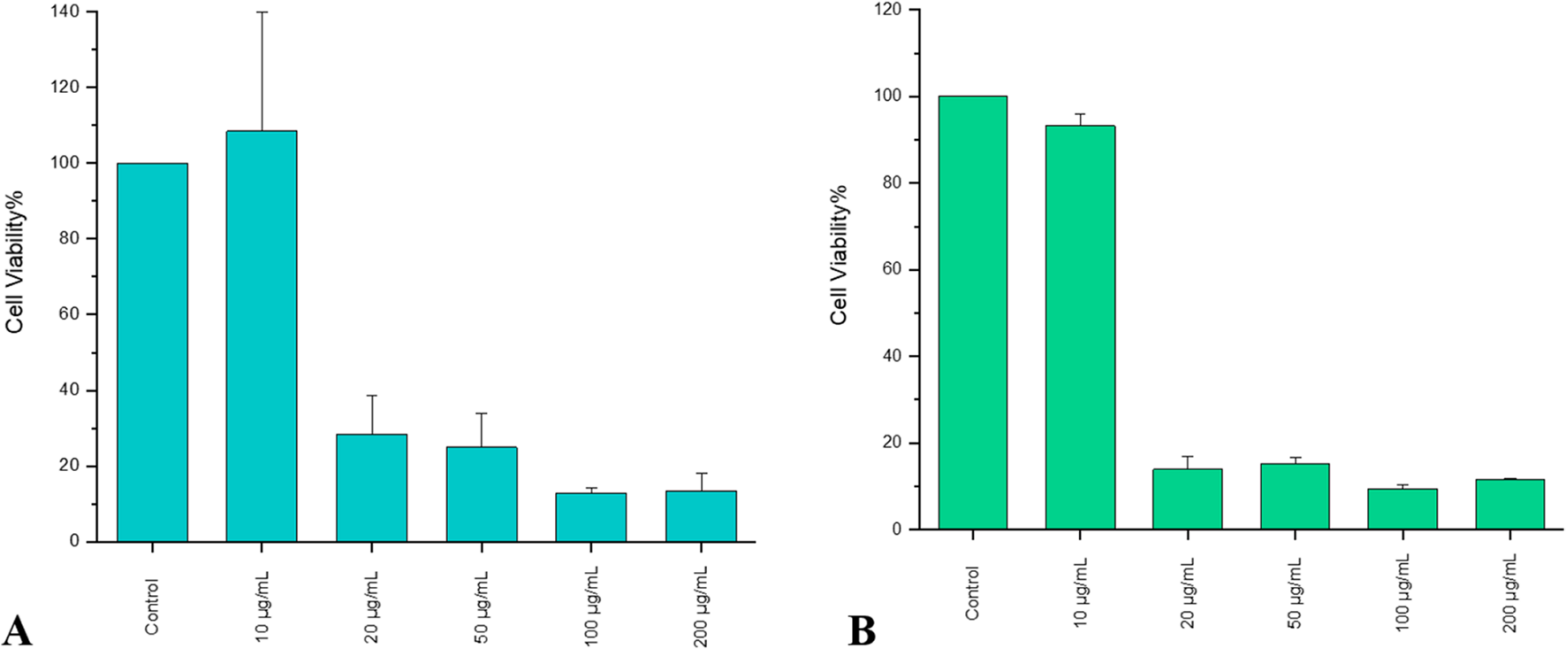
Shows the cytotoxicity effect of biofilm-AgNPs with various concentrations where (**A**) is MCF7 and (**B**) is HEPG2 cell lines. The results represent the means of two biological replicates, three replicates each, and the error bars represent the standard error of the mean.

The results indicated that whenever biofilm-AgNPs are used with low concentrations, 8 µg/mL, on a normal HSF cell line, they do not reduce the viability of the cells, as shown Fig. 12. These results are compatible with the results of adding biofilm-AgNPs with low concentration, 10 µg/mL, on cancer cell lines, MCF-7 and HepG2 cell lines, they do not reduce the viability of both cell lines as shown in Fig. 14. It was illustrated in Fig. 14 that using biofilm-AgNPs with higher concentrations reduces the viability of the cells to about 20–30%. Their effect on cancer cell lines, as shown in Fig. 14, are similar to other biosynthesized AgNPs, such as biosynthesized AgNPs using *Bacillus* sp, which induced the apoptosis on MCF-7 by 61% at 50 μg/mL^134^. Another study illustrated the cytotoxicity effect of the dragon fruit peel aqueous extract fabricated AgNPs on HEPG2 cell lines with (IC_50_) 38 μg/mL^135^.

These previously mentioned results could indicate a variety of things. The results represented the effective synergistic effect of using biofilm-AgNPs in combination with ZCSE9 to kill *S. enterica* with a concentration of 3.1 µg/mL. The concentration of 3.1 µg/mL, as demonstrated previously in Fig. 12, can be effective while not reducing the viability of the normal mammalian cells. Moreover, using biofilm-AgNPs with a concentration of 6.25 µg/mL to inhibit *E. coli*, and *S. aureus*. In this study, it is suggested that biofilm-AgNPs can be used in treating *S. enterica, E. coli*, and *S. aureus* infections caused by contaminated food, water, and hands^136–138^. This study demonstrates that it is unlikely to treat *P. aeruginosa* and *Bacillus cereus* infections because they require higher concentrations of biofilm-AgNPs to be inhibited, which reduces the viability of normal human cells and cancerous cell lines, as shown in Fig. 14. Nevertheless, biofilm-AgNPs show promising results in treating several bacterial infections rather than using antibiotics or phages alone. In addition, they have high effectiveness and antibacterial function with low concentration, which can be used as a disinfectant agent or topical treatment. They cannot be used to treat cancerous cells because they need higher concentrations, which will be toxic to normal cells, as IC_50_ values were 11.3 and 18.7 μg/mL against MCF7 and HEPG2 cell lines, respectively. In the end, it is suggested that further investigations are needed to identify the effect of combining biofilm-AgNPs with phages specific to other bacteria.

## Conclusion

In this study, and for the first time, the biofilm was utilized to generate biosynthesis AgNPs and acts as a capping and stabilizing agent for the formation process. UV-vis spectroscopy, FT-IR, SEM-EDX and TEM were conducted as characterization analyses to confirm the formation of spherical biofilm-AgNPs with size range < 100 nm and bind to bacterial ghosts. Elemental analysis of synthesized NPs by EDX illustrates that the percentage of silver  > 54% with the presence of organic elements. biofilm-AgNPs can be considered as one of the novel approaches as an alternative to antibiotics and other treatments to reduce MDR bacterial infections, especially those that are initiated from surfaces of hospitals. In addition, using it with a low concentration, 8 µg/mL, could not reduce the viability of mammalian cells. Furthermore, it gives more promising, synergistic results when combined with ZCSE9, where bacteria are inhibited and killed before developing resistance to the phage. Eventually, this work opens the door for using the phages with metallic nanoparticles as a combination with synergistic activity.

## Data Availability

Data will be made available on request.
